# SVCT2 Overexpression and Ascorbic Acid Uptake Increase Cortical Neuron Differentiation, Which Is Dependent on Vitamin C Recycling between Neurons and Astrocytes

**DOI:** 10.3390/antiox10091413

**Published:** 2021-09-03

**Authors:** Katterine Salazar, Francisca Espinoza, Gustavo Cerda-Gallardo, Luciano Ferrada, Rocío Magdalena, Eder Ramírez, Viviana Ulloa, Natalia Saldivia, Ninoschka Troncoso, María José Oviedo, María José Barahona, Fernando Martínez, Francisco Nualart

**Affiliations:** 1 Laboratory of Neurobiology and Stem Cells, NeuroCellT, Department of Cellular Biology, Faculty of Biological Sciences, University of Concepcion, Concepción 4030000, Chile; katterinsalazar@udec.cl (K.S.); franespinoza@udec.cl (F.E.); rmagdalena@udec.cl (R.M.); edramirez@udec.cl (E.R.); vulloa@udec.cl (V.U.); nasaldivia@udec.cl (N.S.); ninotroncoso@udec.cl (N.T.); maoviedo@udec.cl (M.J.O.); mariajobarahona@udec.cl (M.J.B.); femartin@udec.cl (F.M.); 2 Center for Advanced Microscopy, CMA BIO BIO, University of Concepcion, Concepción 4030000, Chile; guscerda@udec.cl (G.C.-G.); luferrada@udec.cl (L.F.)

**Keywords:** SVCT2, vitamin C, ascorbic acid, dehydroascorbic acid, cortical neurons, neurospheres, vitamin C recycling, neuronal branching, neurites, SIM superresolution, bystander effect

## Abstract

During brain development, sodium–vitamin C transporter (SVCT2) has been detected primarily in radial glial cells in situ, with low-to-absent expression in cerebral cortex neuroblasts. However, strong SVCT2 expression is observed during the first postnatal days, resulting in increased intracellular concentration of vitamin C. Hippocampal neurons isolated from SVCT2 knockout mice showed shorter neurites and low clustering of glutamate receptors. Other studies have shown that vitamin C-deprived guinea pigs have reduced spatial memory, suggesting that ascorbic acid (AA) and SVCT2 have important roles in postnatal neuronal differentiation and neurite formation. In this study, SVCT2 lentiviral overexpression induced branching and increased synaptic proteins expression in primary cultures of cortical neurons. Analysis in neuroblastoma 2a (Neuro2a) and human subventricular tumor C3 (HSVT-C3) cells showed similar branching results. SVCT2 was mainly observed in the cell membrane and endoplasmic reticulum; however, it was not detected in the mitochondria. Cellular branching in neuronal cells and in a previously standardized neurosphere assay is dependent on the recycling of vitamin C or reduction in dehydroascorbic acid (DHA, produced by neurons) by glial cells. The effect of WZB117, a selective glucose/DHA transporter 1 (GLUT1) inhibitor expressed in glial cells, was also studied. By inhibiting GLUT1 glial cells, a loss of branching is observed in vitro, which is reproduced in the cerebral cortex in situ. We concluded that vitamin C recycling between neurons and astrocyte-like cells is fundamental to maintain neuronal differentiation in vitro and in vivo. The recycling activity begins at the cerebral postnatal cortex when neurons increase SVCT2 expression and concomitantly, GLUT1 is expressed in glial cells.

## 1. Introduction

In recent years, it has been postulated that vitamin C has a functional role in neuronal and progenitor cell differentiation [1,2,3,4,5,6,7]. Sodium-dependent vitamin C transporter 2 (SVCT2) has been detected in neurons of the cerebral cortex, cerebellum, hippocampus, entorhinal cortex and hypothalamus [1,3,8,9,10,11]. SVCT2 has also been detected in glial cells, such as marginal astrocytes, microglia, choroid plexus epithelial cells and tanycytes [9,11,12,13,14]. During cerebral cortex development, SVCT2 was detected in radial glia cells with apical polarization; however, low-to-absent expression was observed in embryonic neuroblasts located in the cerebral cortex [2]. SVCT2 knockout animals die immediately after birth, presenting serious defects in the cerebral cortex [15]. Recent evidence has also shown that vitamin C has epigenetic effects on stem cells, stimulating pluripotency through Nanog activation [16]. In addition, another study suggests that SVCT2 activates the JAK/STAT pathway while incorporating vitamin C into cells [17]. Activation of the JAK/STAT pathway by ascorbic acid (AA) promotes the elimination of reactive oxygen species (ROS), epigenetic regulation, stem cell pluripotency and neuronal differentiation [17]. Thus, vitamin C plays a central role in embryonic CNS stem cell and radial glia differentiation.

Unlike neuroblasts, postnatal neurons increase SVCT2 expression [3]. Therefore, vitamin C may participate in neuronal differentiation, stimulating dendritic arborization and synaptic maturation. Treatment of cortical precursor cells with AA in vitro stimulates neuronal differentiation and increases the frequency and amplitude of excitatory postsynaptic miniature currents, promoting the acquisition of synaptic functions [7]. Studies conducted in cultures of hippocampal neurons from SVCT2 knockout mice observed a lower number of primary dendrites in relation to their controls. In addition, neurons isolated from SVCT2 knockout animals have a lower degree of synaptic maturation [5]. We showed that overexpression of SVCT2 in the neuroblastoma Neuro2a cell line induces numerous microtubule-associated protein 2 (MAP2)-positive processes and filopodia [3]. In cells that overexpress SVCT2, AA induces phosphorylation of ERK1/2 kinases, which are key proteins in the signaling pathways that promote postnatal differentiation and maturation in cortical neurons [3]. Recently, we showed that vitamin C actively stimulates neurite formation in SVCT2-positive neurospheres, with even greater activity than retinoic acid (Espinoza et al., 2021), a molecule widely used to induce neuronal differentiation. Despite the evidence that SVCT2 and AA are important molecules in neuronal arborization and synaptic maturation [3,5,8], to date there is no report that demonstrates this assumption in cortical neurons overexpressing SVCT2.

It is also important to consider that the cellular effects of vitamin C in the brain are produced at concentrations of extracellular vitamin C >200 µM, which allow an accumulation of intracellular vitamin C up to 10 mM [18]. Under these conditions, neuronal viability is dependent on the presence of vitamin C recycling cells, such as astrocytes, which are capable of recycling AA from dehydroascorbic acid (DHA), which promotes metabolic modifications and neuronal death [19,20]. In an oxidative environment, DHA-induced death is due to necroptosis [21]. Intracellularly, we cannot rule out that DHA is also transported to the mitochondria or within the endoplasmic reticulum by GLUTs, reducing the possibility of inducing cell death [22].

In the present work, we report that SVCT2 overexpression increases neuronal arborization in cultures of cortical neurons, Neuro2a and HSVT-C3 cells, with the genesis of primary, secondary and tertiary neuritic processes and the presence of numerous PSD-95-positive dendritic spines in neurons, which establish synaptic contact with Piccolo-positive terminals. Additionally, we observe that neurite formation is dependent on DHA recycling by glial cells. In vitro analysis with a selective GLUT1-glial inhibitor strongly decreases neurite formation. Similarly, by injecting the GLUT1 inhibitor at the cortical tissue in situ, during postnatal differentiation of the cerebral cortex, neurite length and arborization decreases. Our study proposes that vitamin C and SVCT2 expression are important in neuronal postnatal differentiation, a process that in turn depends on the recycling of vitamin C between astrocytes and neurons.

## 2. Materials and Methods

### 2.1. Animals

The animal care procedures were performed in accordance with the “Manual de Normas de Bioseguridad” (Comisión Nacional de Ciencia y Tecnología, CONICYT) and the Animal Care and Use Committee of the University of Concepcion (Concepción, Chile). The experimental protocols were approved by the Concepción University Licensing Committee, grant number ethic code 1181243. The animals were housed under a 12 h light/dark cycle with food and water available ad libitum.

### 2.2. Primary and Cell Line Culture

Primary cultures of neurons and neurospheres were obtained from 17-day-old Sprague Dawley rat embryos. For neurons, forebrains were digested for 10 min at 37 °C with 0.25% (*w*/*v*) trypsin and 0.2% (*w*/*v*) EDTA in 0.1M phosphate buffer (pH 7.4, 320 mOsm) and triturated to homogeneity in MEM supplemented with 10% fetal bovine serum, 2 mM glutamine, 100 U/mL penicillin, 10 µg/mL streptomycin and 2.5 µg/mL fungizone. The cells were plated at a density of 2.6 × 10^5^ cells/cm^2^ or 1 × 10^5^ cells/cm^2^ (for recycling experiments) onto glass coverslips coated with poly-L-lysine (Sigma, St Louis, MO, USA). After 30 min, non-adhered cells were removed, and the attached cells were cultured with Neurobasal media (Gibco Co., Rockville, MD, USA) supplemented with B27 (Gibco Co., Rockville, MD, USA), 2 mM glutamine, 100 U/mL penicillin, 10 µg/mL streptomycin and 2.5 µg/mL fungizone for 3 days. The cultures were maintained in an incubator at 37 °C, 5% CO_2_ and 95% humidity. After 3 days, the cells were transduced with the lentiviral particles, hSVCT2-EYFP or EGFP as described by Salazar et al. [3]. The cortical neuron cultures were incubated with conditioned medium containing the lentivirus (dilution 1:1) at 37 °C for 14–16 h. The transduced cells were maintained in culture for 24, 48, 72 and 96 h.

For neurospheres cultures, the cerebral cortex was dissected and mechanically disaggregated in neural stem cell (NSC) proliferation medium (Stem Cell Technologies, Vancouver, BC, Canada) supplemented with epidermal growth factor (EGF; 20 ng/mL), fibroblast growth factor (FGF; 10 ng/mL), and heparin (10 ng/mL) (Stem Cell Technologies). The cellular suspension was seeded in T25 cell culture flasks (BD Falcon^TM^, Franklin Lakes, NJ, USA) at approximately 100,000 cells/cm^2^ to allow for neurosphere formation. After 2 days in vitro (DIV), the neurospheres were collected and adhered to poly-L-lysine-coated dishes (0.1 mg/mL poly-L-lysine; Sigma-Aldrich, St. Louis, MO, USA). Primary astrocyte cultures were obtained from the forebrains of 1- to 4-day-old postnatal Sprague–Dawley rats using standard protocols [19]. The U87 cell line was maintained in DMEM supplemented with 10% FBS medium. For coculture assays, U87cells were added to 12-well plates with adhered neurospheres at a density of 25,000 cells/cm^2^. Finally, Neuro2a cells were cultured in DMEN/F12 [3] and HSVT-C3-subventricular malignant glioma cells were grown in DMEM/F12 media [23].

### 2.3. RT-PCR and Quantitative RT-PCR (QRT-PCR) Analyses

Non-treated and transduced cortical neurons cultures from 12-well plates were used to prepare total RNA extracts purified with Trizol reagent (Invitrogen, Carlsbad, CA, USA) and quantified in a SmartSpec300 spectrophotometer (Bio-Rad, Hercules, CA, USA). For RT-PCR, 1 μg of RNA was pre-treated with DNase I (Fermentas, Burlington, ON, Canada), processed and underwent thermocycling as described in Salazar et al. [8]. QRT-PCR analyses were performed using a Brilliant SYBR Green QPCR master mix kit (Stratagene, Cedar Creek, TX, USA) in a final volume of 20 μL containing 2 μL of cDNA (obtained from 250 ng total RNA), 500 nM of each primer, 4 mM MgCl2, and 2 µL of Brilliant SYBR Green QPCR master mix (Stratagene). PCR reactions were carried out in an MX3005P thermocycler (Stratagene) using the following cycling conditions: one cycle at 95 °C for 10 min followed by 40 cycles at 95 °C for 10 s, 55 °C for 5 s and 72 °C for 10 s. The following sets of primers were used: GAPDH (NM_017008.4), forward 5′-GCA AGT TCA ACG GCA CAG TCA AG-3′ and reverse 5′-CGT GGT TCA CAC CCA TCA CAA AC-3′; alpha synuclein (NM_019169.3), forward 5′-AGG GAG TCG TTC ATG GAG TG-3′ and reverse 5′-TTC CAG GAT TCC CTC TTG TG-3′; rabphilin 3a (NM_133518.2), forward 5′-GTC AAG CTC TGG CTG AAA CC-3′ and reverse 5′-GCA GCC TCC GAT GTA ATC AT-3′; and synaptotagmin VII (NM_021659.2), forward 5′-GAG GTG TCC ATC CCT CTC AA-3′ and reverse 5′-AGC CAC ACC TTC ACA TAG GG-3′. The primers used to detect SVCT2 mRNA expression were the same as those used for non-quantitative [8]. To verify the results, all experiments were performed in triplicate. The relative expression of SVCT2 mRNA to GAPDH mRNA was calculated using the 2^−ΔΔCt^ method.

### 2.4. Immunocytochemistry 

Cortical neurons, Neuro2a cells and HSVT-C3 cells cultures were grown on coverslips, fixed with 4% paraformaldehyde for 30 min at 22 °C, washed with phosphate-buffered saline Tris-phosphate buffer [24,25], and incubated in Tris-phosphate buffer containing 1% bovine serum albumin (BSA) and 0.2% triton X-100 for 5 min at 22 °C. The cells were incubated overnight at 22 °C with the following antibodies: anti-SVCT2 (1:100; Santa Cruz Biotechnology, Santa Cruz, CA, USA), anti-MAP2 (1:50; Chemicon, Temecula CA, USA), anti-MAP1B (1:250; Santa Cruz Biotechnology), anti-Piccolo (1:1000; Synaptic System, Goettingen, Germany) or anti-PSD95 (1:500; monoclonal; UC Davis/NIH NeuroMab Facility, Davis, CA, USA). The cells were then incubated for 2 h with Cy3-conjugated affinity-purified donkey anti-goat IgG, Cy3-conjugated affinity-purified donkey anti-rabbit IgG or Cy3-conjugated affinity-purified donkey anti-mouse IgG (all 1:200; Jackson ImmunoResearch, West Grove, PA, USA) at 22 °C. In addition, the cells were incubated with Hoechst 33342 (1:1000; Invitrogen, Waltham, MA, USA) for nuclear staining. Spectral confocal microscopy images were obtained using a Zeiss confocal microscope model LSM780, equipped with DPSS laser (405 nm), argon laser (458–488–514 nm), DPSS laser (561 nm) and helium laser-neon (633 nm). The structured illumination microscopy-superresolution (SIM-SR) microscopy images were obtained in the Zeiss SIM-SR equipment, model Elyra S1, which is equipped with DPSS diode laser (488–561 nm) and two lenses: a 10×/0.3 NA lens and a 63×/1.4 NA. The two-dimensional reconstructions in the xz and yz planes and the three-dimensional projections were made with the Zen program V7.0.7.2 (Carl Zeiss AG, Berlin, Germany) or with the Imaris Cell Imaging Software 7.4 (BitPlane AG, Belfast, UK). To specifically localize the RE, CellLightTM ER-RFP BacMam 2.0 (Molecular Probes, Waltham, MA, USA) baculovirus was incubated for 24 h in HSVT-C3 cells. To calculate the percentage of neurospheres with neurites in one sample, 45–50 neurospheres were analyzed per condition; one neurite was considered a process positive for βIII tubulin longer than 10 µm (diameter of one cell into the neurosphere).

### 2.5. Sholl Analysis

The images from which the quantification was obtained in an Eclipse Ti epifluorescence microscope (Nikon Instrument Inc, New York, NY, USA). The quantification was carried out following the Sholl method [26] added in the reference list, which is based on counting the number of intersections of the processes in a successive circumferential radius of 10 µm each one with center in the cellular soma.

### 2.6. Vitamin C Uptake

Cortical neurons transduced with hSVCT2-EYFP or EGFP lentivirus were carefully selected under the microscope to ensure that only plates with uniformly growing cells were used. Additionally, HSVT-C3 were analyzed. Briefly, the cells were washed with incubation buffer (15 mM HEPES, 135 mM NaCl, 5 mM KCl, 1.8 mM CaCl_2_ and 0.8 mM MgCl_2_), or a buffer in which NaCl was replaced by choline chloride when indicated, and incubated in the same medium for 10 min at 37 °C. Uptake assays were performed in 500 μL of incubation buffer containing a final concentration of 100μM l-^14^C-L-AA (specific activity of 4 mCi/mmol; DuPont-NEN, Boston, MA, USA) and 0.1 mM dithiothreitol (DTT). The uptake was stopped by washing the cells twice with an ice-cold incubation buffer consisting of 0.8 mM HgCl_2_. The cells were homogenized in 0.5 mL of lysis buffer (10 mM Tris–HCl, pH 8.0 and 0.2% SDS) and assayed by liquid scintillation spectrometry to quantify the incorporated radioactivity in counts per min (CPM).

### 2.7. Cell Viability Using XTT and IncuCyte^®^ Analysis

Cell Proliferation kit (XTT) (BI Biological Industries, Galilee, Israel) was used to measure viability. Thus, 500 µL of proliferation medium containing 2DIV neurospheres were seeded in 24-well plates previously treated with poly-l-lysine. The measurement was carried out at 12 and 72 h post-treatment, following the protocol indicated by the manufacturers. For analysis by IncuCyteS3, neurospheres were seeded in ultra-clear bottom, microscopic grade, 24-well plates in the presence of the SYTOX^®^ Green probe (1×, Invitrogen). The images were acquired in the IncuCyte^®^S3 equipment (Sartorius GA, Gotinga, Germany), which was configured to take four photos for each well with a 10× magnification objective, each 1 h, for a time period of 72 h. The images were processed using the IncuCyte S3 software and the basic module, which allows the analysis of the number of objects that present green fluorescence with respect to the total number of cells identified by phase contrast.

### 2.8. In Vivo Tests

In vivo studies were carried out in rats at postnatal day 5, which were subjected to an injection in the outer zone of the cerebral cortex. In total, 4 µL of the lentivirus EGFP (control) or lentivirus-EGFP mixed (1:1 ratio) with the GLUT1 inhibitor, WZB117 (Sigma Aldrich, San Luis, MO, USA), was injected. The animals were anesthetized for 3 min on ice, then the injection was performed by tracing an imaginary line between the left eye and the intersection of the sagittal and coronal sutures, a point known as “Bregma”. The midpoint of this line was searched, puncturing 1.5 mm deep. They were monitored for 72 h post-injection following the postnatal animal monitoring and end-point procedure. After 72 h, on day P8, the brain was extracted for analysis.

### 2.9. Golgi–Cox Impregnation

Ultra-rapid Golgi stain (URG) was performed following the protocol published by [27]. P8 rat brains were obtained and subsequently fixed by vascular perfusion with 4% PFA. Additionally, URG impregnation was performed on 150 µm sections obtained from the same brains. The sections were washed in 1 × PBS and then incubated in URG solution (5% *w*/*v* Mercury chloride, 5% *w*/*v* Potassium dichromate and 1.6% *w*/*v* Potassium chromate) for 36 h to 37 °C. Washes were carried out with distilled H_2_O and subsequently incubated with 10% *v*/*v* ammonium hydroxide for 20 min at room temperature and darkness. Finally, tissues were incubated for 20 min with 10% *w*/*v* sodium thiosulfate, and two washes were carried out with distilled H_2_O. Finally, the sections were mounted on slides previously covered with gelatin, dried in the dark for 15 min, and a mounting medium for fluorescence (Dako, Santa Clara, CA, USA) was used.

### 2.10. Statistical Analysis

The data represent the mean ± SD with three independent experiments obtained from three independent biological samples. Statistical analyses were performed using GraphPad Prism version 6.01 (GraphPad Software, San Diego, CA, USA).

For qRT-PCR, the data were processed using Student’s t-tests with Welch correction. ** *p* < 0.01; *** *p* < 0.001. For Sholl evaluation, statistical studies were carried out using analysis of variance (ANOVA, followed by Bonferroni post-test). ** *p* < 0.01, *** *p* < 0.001 were considered to be statistically significant. For vitamin C uptake, data were processed by Student’s T-tests with Welch’s correction. * *p* < 0.05; ** *p* < 0.005; *** *p* < 0.0005.

## 3. Results

### 3.1. SVCT2 Lentiviral Transduction Increases AA Uptake and Cell Arborization

**Figure 1:**
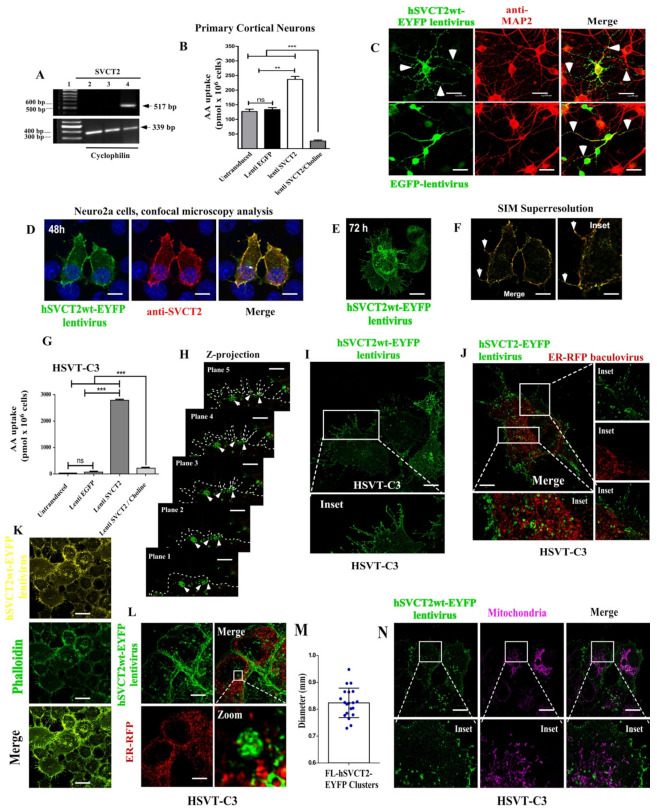
SVCT2 overexpression increased cellular branching. (**A**) RT-PCR analysis of SVCT2 in mRNA isolated from nontransduced (lane 2) and EGFP- or hSVCT2wt-EYFP-overexpressing neurons (lanes 3 and 4, respectively). The 100 bp standard (lane 1). RT-PCR analysis of cyclophilin was performed as an internal control. (**B**) Uptake of 100 μM AA was analyzed in the presence of NaCl or choline at 37 °C in nontransduced and EGFP- or hSVCT2wt-EYFP-overexpressing neurons. (**C**) Immunofluorescence analysis with an anti-MAP2 antibody (Cy3, red channel) in EGFP- or hSVCT2wt-EYFP- overexpressing neurons (green channel). Both lentiviruses transduced MAP-positive cells; however, SVCT2 overexpression increased the number of neuronal processes in vitro (arrows). (**D**,**F**) SVCT2 overexpression in Neuro2a cells analyzed by confocal and SIM superresolution microscopy. hSVCT2-EYFP lentivirus expression (green channel) and anti-SVCT2 detection (red channel). Overexpressed SVCT2 colocalized with anti-SVCT2 antibodies (yellow in merge image) and induced neurite-like processes (arrows). After 72 h of SVCT2 expression, neurites were clearly observed in transduced cells (**E**). (**G**–**N**) SVCT2 overexpression in HSVT-C3 cells. (**G**) Uptake of 100 μM AA was analyzed in the presence of NaCl or choline at 37 °C in nontransduced and EGFP- or hSVCT2wt-EYFP-overexpressing HSVT-C3 cells. (**H**) Consecutive z-planes of C3 cells overexpressing SVCT2. The overexpressed protein appeared to be transported in vesicle-like structures (arrows) that fused to the cell membrane and reached the thin filopodia processes (**I** and inset). (**J**) The intracellular vesicles had partial colocalization with the RER marker (calreticulin-KDEL-RFP bacculovirus, red-channel) (**J** and inset); however, the filopodia and bleb-like and ruffes-like structures were positive for phalloidin (**K**). (**L**–**M**) RER (RE-RFP+) and SVCT2-EYFP localization to analyze the size distribution of multivesicular structures (zoom), quantified in the (**M**) graph. (**N**) Distribution of mitochondria (Mitotracker-positive) and SVCT2-positive cell structures, where the absence of colocalization was observed. The data represent the means ± SD of three experiments. *** *p* < 0.001, ** *p* < 0.01, ns = not significant. Scale bars in (**C**), 20 μm; in (**D**,**E**), 15 μm; in (**F**,**K**), 10 μm; in (**H**), 1 μm; in (**I**–**J**,**L**,**N**), 5 μm.

In order to determine the effect of SVCT2 overexpression on the morphology of cortical neurons in vitro, we performed lentiviral hSVCT2wt-EYFP transductions. In experiments carried out at 48 h post-transduction, hSVCT2wt-EYFP mRNA expression was confirmed by RT-PCR, amplifying a 517 bp fragment with specific primers only for the human sequence (Figure 1A, lane 4). In contrast, no hSVCT2wt-EYFP mRNA expression was detected in the non-transduced cultures (Figure 1A, lane 2) or EFGP-transduced neurons (Figure 1A, lane 3). [^14^C]-AA uptake analysis demonstrated the functionality of the expressed transporter as the cultures of neurons transduced with hSVCT2wt-EYFP lentivirus captured twice the amount of AA (236.74 ± 20.58 pmol × 10^6^ cells) as compared to nontransduced neurons (127.13 ± 13.75 pmol × 10^6^ cells) or those transduced with the EGFP lentivirus (133.58 ± 11.80 pmol × 10^6^ cells) (Figure 1B).

Using confocal microscopy, we observed that neurons transduced with hSVCT2wt-EYFP lentivirus were also MAP2-positive and developed a greater degree of cellular arborization with the extension of primary/secondary processes and small filopodia (Figure 1C). These results suggest that functional SVCT2 overexpression in cortical neurons increases the morphological differentiation of cortical neurons in vitro. To analyze whether the effect observed in primary neurons can be reproduced in other types of neural cells, we overexpressed SVCT2 in Neuro2a (Figure 1D–F) and HSVT-C3 cells (Figure 1G–M), which have characteristics of neural stem cells [23]. In both cell lines, hSVCT2-EYFP was preferentially detected at the cell membrane after 48 h overexpression, which was also demonstrated in Neuro2a cells with immunofluorescence analysis using anti-SVCT2 (Figure 1D, red channel and merge image) using confocal microscopy and SIM superresolution analysis (Figure 1F). After 72 h of SVCT2 overexpression, Neuro2a cells clearly increased their processes and filopodia (Figure 1E). In HSVT-C3 cells, we also found that SVCT2 overexpression consistently increased AA uptake at 5 min in cells that showed low endogenous AA uptake (30.2 ± 0.37 pmol × 10^6^ cells in untransduced cells). However, in cells transduced with hSVCT2wt-EYFP lentivirus, the uptake was 2755.7 ± 63.5 pmol × 10^6^ cells, compared with 50.4 ± 0.96 pmol × 10^6^ cells in those transduced with the EGFP lentivirus (Figure 1G) or 205.8 ± 381 pmol × 10^6^ cells in hSVCT2wt-EYFP lentivirus-transduced cells incubated with AA + choline.

After SVCT2 overexpression, vesicular complexes were detected near the cell membrane (Figure 1H,I), with restricted SVCT2 colocalization with RER cisterns (calreticulin-KDEL-RFP bacculovirus labeling) (Figure 1J,L), which may indicate that this transporter is rapidly synthesized and transported to the cell membrane. In the cell membrane, it is located in neurites that are filopodia-phalloidin-positive (Figure 1K) and in bleb-like and ruffes-like structures (Figure 1L and insets). In some regions of the cells (neurite formation zones), vesicular-like structures were observed in juxtaposition to the cell membrane (Figure 1L), with extensive size distribution (Figure 1M). Finally, overexpressed hSVCT2-EYFP was not localized in the mitochondria (Figure 1N), which were identified with mitotracker (violet channel).

### 3.2. SVCT2 Overexpression in Cortical Neurons Increased Arborization, Synaptic Gene Expression and the Presence of Dendritic Spine-Like Structures

**Figure 2:**
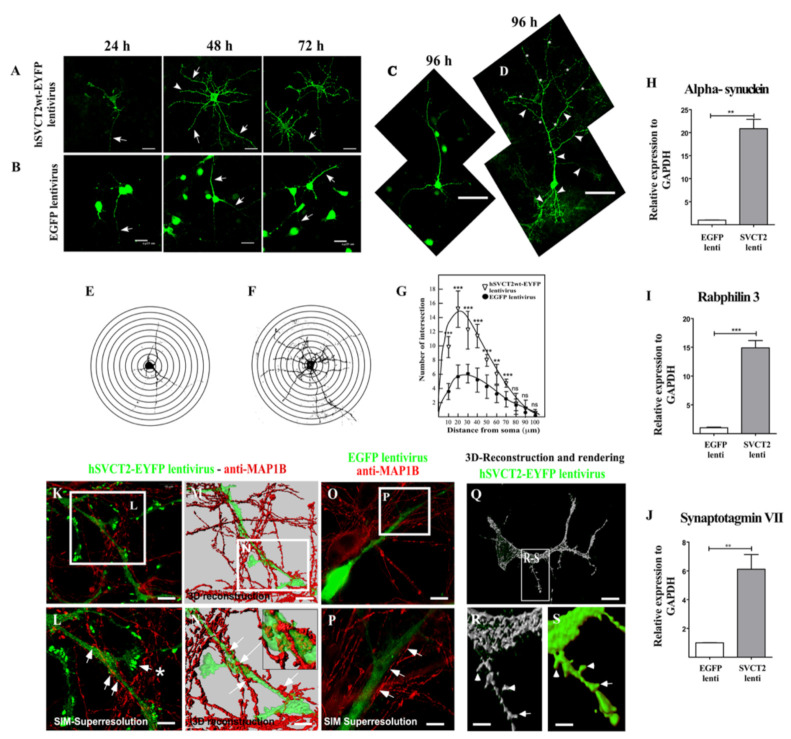
SVCT2 overexpression in cortical neurons promoted arborization and synaptic gene expression. (**A**,**B**) Cortical neurons overexpressing hSVCT2wt-EYFP-A or EGFP-B for 24, 48 and 72 h in vitro. Some cellular processes were indicated with arrows. (**C**,**D**) Comparative images between EGFP- or hSVCT2wt-EYFP-overexpressing cortical neurons at 96 h, respectively. (**E**,**F**) Representation and Sholl analysis of EGFP- or hSVCT2wt-EYFP-overexpressing cortical neurons at 48 h, applying digitally concentric rings spaced 10 µm apart centered on the soma center to a radius of 100 µm. (**G**) Sholl analysis plot of EGFP- or hSVCT2wt-EYFP-overexpressing cortical neurons at 48 h. (**H**–**J**) QRT-PCR analysis of the alpha-synuclein, rabphilin 3 and synaptotagmin VII mRNA levels in samples isolated from EGFP- or hSVCT2wt-EYFP-overexpressing cortical neurons at 48 h. ** *p* < 0.01, *** *p* < 0.001. Results are mRNA levels relative to GAPDH and represent the mean ± SD for three independent experiments. (**K**,**L**) Confocal microscopy (**K**) or superresolution analysis (**L**) of neurons transduced with hSVCT2-EYFP lentivirus and incubated with anti-MAP1B. (**M**,**N**) 3D reconstruction and rendering of the green channel using Imaris 7.4 software (Bitplane) in Z-stack of SIM superresolution images. The presence of numerous dendritic spines of the mushroom type (arrows and asterisk) and thin dendritic spines (arrowheads) was observed (**Q**–**S**). (**O**,**P**) Confocal microscopy (**O**) or superresolution analysis (**P**) of neurons transduced with EGFP-lentivirus and incubated with anti-MAP1B antibodies. Neurites without thin dendritic spines (arrows) were observed. The data represent the means ± SD of three experiments. *** *p* < 0.001, ** *p* < 0.01. Scale bars in (**A**,**B**), 21 μm; in (**C**,**D**), 45 μm; In (**K**,**M**,**O**,**Q**), 10 μm; in (**L**,**M**,**P**), 5 μm; and (**R**,**S**), 2 μm.

Cortical neurons were fixed and analyzed by confocal microscopy at 24, 48, 72 and 96 h post-transduction with hSVCT2wt or EGFP lentivirus (Figure 2A,B). Unlike control neurons (Figure 2B), there was an increase in the arborization of cortical neurons that overexpressed hSVCT2wt-EYFP that increased over time following lentiviral transduction (Figure 2A, arrows). At 96 h, large cells with long primary, secondary and tertiary processes and short filopodia-like processes with dendritic spines were observed (Figure 2D, arrowheads). In order to quantify the differences in arborization between control cultures and those expressing SVCT2 at 48 h post-transduction, the Sholl method was used (Figure 2E–G). As shown in Figure 2G, there were significant differences in the number of intersections between neurons treated with the different lentiviruses. Thus, overexpression of hSVCT2 accelerates the differentiation of cortical neurons in vitro.

To study whether SVCT2 and vitamin C may regulate genes associated with synaptic connectivity, we evaluated whether there was an increase in mRNA expression of genes related to synaptic maturation, such as alpha-synuclein, rabphilin 3 and synaptotagmin VII. Our results showed an almost 21-fold increase in alpha-synuclein expression in neurons that overexpressed SVCT2 (20.86 ± 4.03) (Figure 2H), a 15-fold increase in rabphilin expression in neurons that overexpressed SVCT2 (14.90 ± 2.84) (Figure 2I), and a 7.6-fold increase in the expression of synaptotagmin VII in neurons overexpressing SVCT2 (17.61 ± 1.20) relative to the control (Figure 2J).

Next, we evaluated the presence of dendritic spine-like structures in neurons that overexpressed SVCT2 by SIM-SR and 3D reconstruction rendering analysis. Only dendritic-like processes were SVCT2-positive; the axons were detected using anti-MAP1b antibodies (Figure 2K–N). Axons were detected by establishing an “en-passant” relationship with the dendritic-like structures (Figure 2M,N). In different regions, the axons formed synaptic-like contacts, which were observed in greater detail in Figure 2N. High-magnification and 3D-rendering analysis showed small dendrite-like membrane protrusions with thin and mushroom shapes (Figure 2Q–S, arrowheads and arrows, respectively). These results suggest that SVCT2 and vitamin C promote the functional maturation of cortical neurons in vitro.

### 3.3. High-Resolution Imaging Confirmed the Presence of SVCT2 in the Cell Membrane of Differentiated Neurons In Vitro and Its High Expression in Regions of Synaptic Contacts

**Figure 3:**
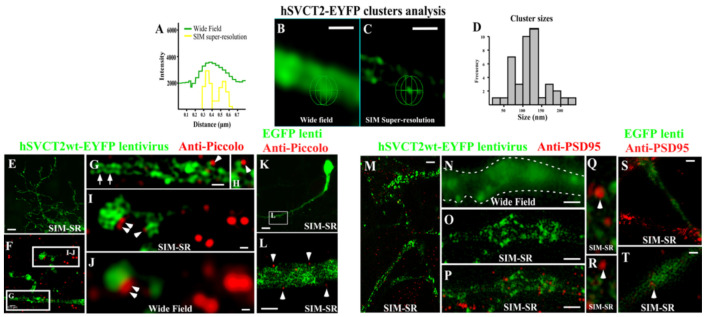
SVCT2 neuronal clusters are co-distributed with post-synaptic protein. (**A**–**D**) SIM Super-Resolution and Wide Field analysis of hSVCT2-EYFP cluster distribution and size in transduced neurons. (**E**–**L**) SIM Superresolution analysis to detect the colocalization between the green processes of neurons transduced with EGFP (**K**,**L**) or hSVCT2wt-EYFP (**E**–**J**) lentivirus and the piccolo presynaptic protein (red) (**F**–**J**) in cultures transduced for 48 h. (**M**–**T**) SIM Superresolution analysis to detect the colocalization between the green processes of neurons transduced with EGFP (**S**,**T**) or hSVCT2wt-EYFP (**M**–**R**) lentivirus and the PSD95 post-synaptic protein (red) (**O**–**R**) in cultures transduced for 48 h. Processes that overexpressed SVCT2 were also positive for PSD95 and in close proximity with piccolo-positive contacts (arrowheads). SVCT2 clustering on the plasma membranes was observed (arrows). (**S**,**T**) SIM Superresolution analysis to detect the colocalization between the green processes of neurons transduced with EGFP and PSD95 protein (red). Scale bars in (**B**,**C**,**F**–**J**,**L**–**N**,**P**,**S**,**T**), 0.5 μm; (**E**,**K**), 10 μm.

Using SIM-SR imaging, we reconstructed dendritic arborization in in vitro-differentiated neurons that overexpressed SVCT2, at a resolution level of 100 nm (Figure 3A–C,E), which was compared with widefield imaging to define the resolution limit (Figure 3A–C). SVCT2-EYFP was homogeneously distributed in neuronal processes, preferably in the cell membrane that limits the edges of each process (Figure 3C,G). High magnification analysis showed SVCT2 clustering both at the intracellular level and in the cellular membrane (Figure 3C,G). Using SIM-SR, we analyzed the cluster size frequency of SVCT2-positive structures, and the main frequency was detected between 100–150 nm (Figure 3D). Next, we analyzed the distribution of different synaptic proteins, including Piccolo (Figure 3G–J). This pre-synaptic protein was located at focal points along different neurites, where it was common to find a gap between SVCT2-positive dendritic spines and Piccolo-positive synaptic contacts by SIM-SR analysis (Figure 3H–I). Similar results were obtained when analyzing the distribution of SVCT2-EYFP and the post-synaptic protein, PSD95; however, most of the positive signal for PSD95 was found in dendritic projections in regions neighboring SVCT2 localization (Figure 3M–R). Controls with EGFP lentivirus showed a lower level of neurites and immunostaining for Piccolo and PSD95 (Figure 3K–L and Figure 3S–T, respectively). These analyses demonstrate that neuronal SVCT2, with cluster distribution in dendritic spines, co-distributes with post-synaptic protein.

### 3.4. Vitamin C Recycling Maintained Neuritic Processes in Primary Neurons

**Figure 4:**
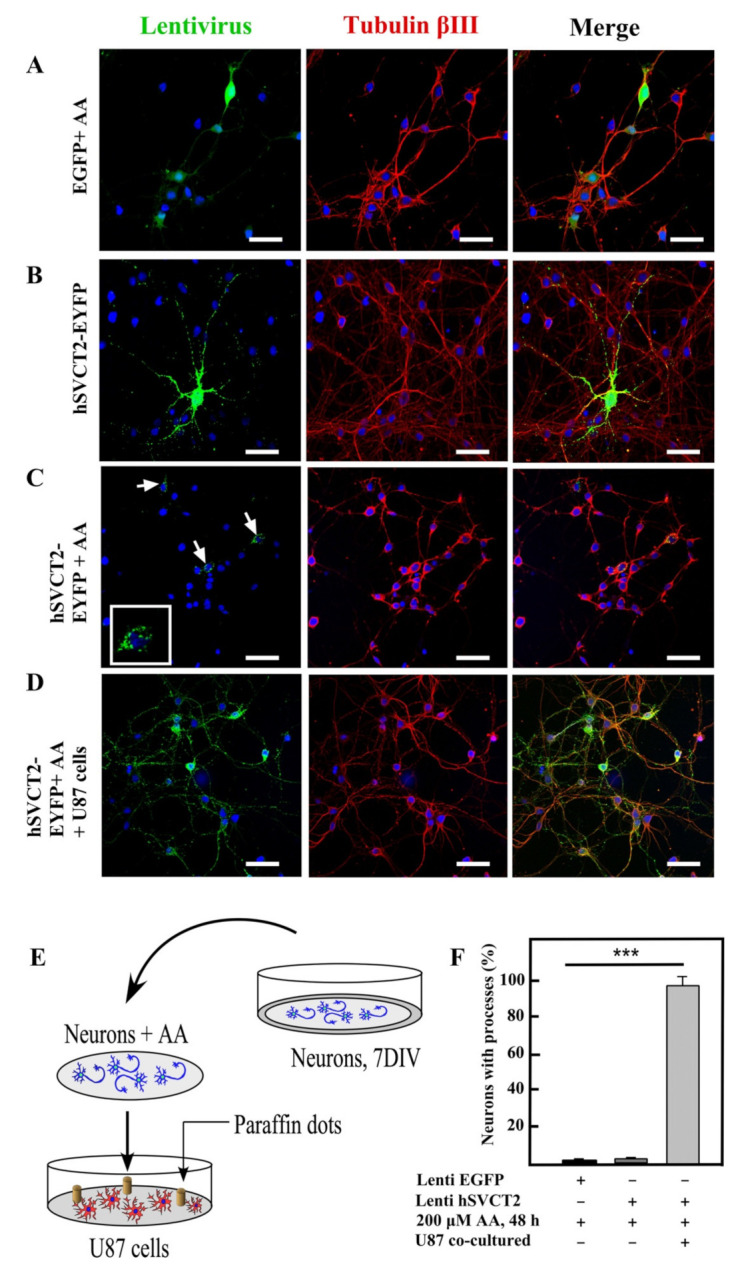
Astrocyte-like cells induced vitamin C recycling and maintain neurite processes in neurons overexpressing SVCT2 and treated with ascorbic acid. (**A**–**D**) Confocal microscopy analysis of hSVCT2wt-EYFP- or EGFP- overexpressing cortical neurons transduced for 48 h in vitro and incubated with anti-tubulin BIII (red). The cells were cultured in the absence or presence of ascorbic acid (AA). (**A**) EGFP-overexpressing cortical neurons treated with AA. (**B**) hSVCT2wt-EYFP-overexpressing cortical neurons. (**C**) hSVCT2wt-EYFP- overexpressing cortical neurons treated with AA. (**D**) hSVCT2wt-EYFP- overexpressing cortical neurons treated with AA and incubated with astrocytes-like cells (U87, recycling cells). (**E**) Scheme of the experimental procedure of co-culture between neurons and astrocytes. (**F**) Neurons cultured in different conditions. Quantification analysis of neurons with processes. The data represent the means ± SD of three experiments. *** *p* < 0.001. Scale bars in (**A**–**D**), 25 μm.

In the mature brain, vitamin C can reach high concentrations in the CSF and cerebral parenchyma [18]. In neurons, vitamin C can reach concentrations of 10 mM; therefore, it is possible that it oxidizes to DHA. DHA regulates neuronal metabolism, inhibiting glycolysis and finally generating cell death [20,28,29]. When neurons increase the expression of SVCT2 and capture high concentrations of vitamin C, they should activate DHA recycling mechanisms within astrocytes. To analyze this hypothesis, we overexpressed hSVCT2-EYFP or EGFP in neuronal cells in vitro to induce dendritic arborization (Figure 4A–F), and then treated the cells with 200 µM AA. After 48 h, we observed the effect generated in the presence or absence of the DHA recycling cells, U87 cells, which are of astrocytic lineage (Figure 4D). Neurons cultured without U87 cells but treated with AA (without DHA recycling) and overexpressing SVCT2-EYFP had a significant shortening of their neuritic processes (Figure 4C,F). In addition, SVCT2 was located intracellularly in the neuronal soma (Figure 4C, inset). However, neurons with DHA recycling (cultured with U87 cells and treated with AA) and overexpressing SVCT2-EYFP maintained their arborization and SVCT2 distribution in all neurites (Figure 4D,F). Thus, DHA recycling was essential to maintain neurite arborization.

### 3.5. The Recycling of Vitamin C Maintained Neuritic Processes in Neurospheres

Before conducting experiments that allow us to analyze the effect of DHA recycling in the cerebral cortex of postnatal rats and in neurites growth, we conducted studies in 3D cultures of neural precursors that form neurospheres. Recently, we showed that neurospheres expressed high levels of SVCT2 and, when treated with AA, actively generated neurites [30]. This approach seemed robust and complementary to SVCT2 overexpression using primary culture of neurons transduced with lentiviruses. The neurospheres treated with AA also produced DHA, which must be recycled to prevent inhibition of neurite growth and fragmentation [30] In this way, we analyzed the in vitro effects of the GLUT1 inhibitor, WZB117, on DHA recycling and neurite genesis, in cocultures of neurospheres and U87 cells (recycling cells), as well as the cell viability of U87, neurons and astrocytes.

**Figure 5:**
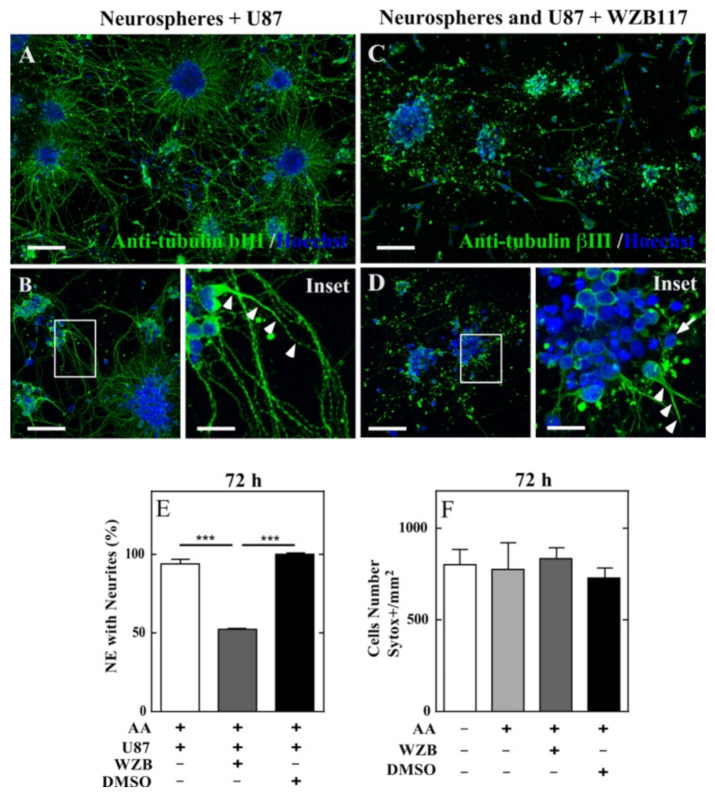
Co-culture of neurosp heres and U87 cells, generation of neurites and effect of the GLUT1 inhibitor, WZB117, on DHA recycling and neurite outgrowth. (**A**–**D**) Immunocytochemical analysis using anti- βIII tubulin (green) and Hoechst for nuclear staining (blue). (**A**) neurospheres and U87 co-cultured cells treated with 100 µM AA for 72 h. (**B**) Magnified image, highlighting the presence of long neuritic processes (Inset). (**C**) Neurospheres and U87 co-cultured cells treated with 100 µM AA for 72 h and incubated with 5 µM WZB117 for 24 and 48 h. (**D**). Magnified image, where short neuritic processes (Inset, arrow heads) or their absence (Inset, long arrow) were observed. (**E**) Quantification of the percentage of neurospheres with neurites, treated with WZB117 or incubated with DMSO. (**F**) SytoxGreen detection by IncuCyte^®^ analysis to determine cell viability (*n* = 3). Scale bar; 20 µm. Statistical analysis ANOVA, followed by Tukey posttest. *** *p* < 0.001. Bar graphs represent mean + SEM. In total, 45–50 neurospheres were quantified by condition. Scale bars, in (**A**,**C**), 200 µm; in (**B**,**D**), 50 µm; in insets, 20 µm.

As has been characterized in detail in Espinoza et al. [30], neurospheres grown in vitro and treated with AA, in the presence of U87 cells (DHA recycling cells), generated neurites efficiently (Figure 5A,B). Thus, similar cultures of neurospheres were co-cultured with U87 cells and treated with 5 µM WZB117 at 24 and 48 h of AA treatment. Immunoreaction against βIII tubulin showed a lower amount of neurospheres with neurites (Figure 5C), compared to the control (Figure 5A). With higher magnification, long neurites from control cultures (Figure 5B, arrowhead) were observed in detail, but not in the presence of WZB117 (Figure 5D). Quantification of the total neurospheres with neurites showed a significant decrease in the presence of the GLUT1 inhibitor (52.36 ± 0.36%), compared to the control (93.94 ± 2.6%) or to supplementation with the vehicle, DMSO (100%) (Figure 5E). The viability of the neurospheres cultures was evaluated during the treatment with AA or WZB117, by incorporating SytoxGreen and analyzed by IncuCyte^®^. No significant differences were detected in the viability of control or treated cultures, ruling out that the lower presence of neurites in the presence of WZB117 could be attributed to a decrease in neurospheres viability (Figure 5F).

### 3.6. GLUT1 Inhibition in U87 Cells, Astrocytes and Neurons Slightly Affected Cell Viability

**Figure 6:**
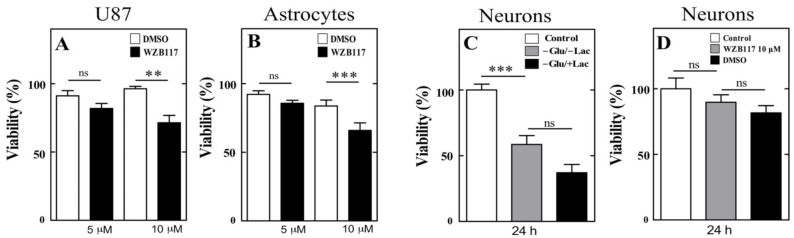
Viability analysis in U87 cells, astrocytes and neurons treated with WZB117, a GLUT1 inhibitor. (**A**,**B**) Cell viability analysis using the XTT method in U87 cells and astrocytes exposed to 5 or 10 µM WZB117 or DMSO. The analyses were carried out after 24 h of incubation with WZB117. (**C**) Viability analysis using the XTT method in 7 DIV cortical neurons incubated with 25 mM glucose (control), without glucose and lactate (−Glu/−Lac) or without glucose and 5 mM lactate (−Glu/+Lac) for 24 h. (**D**) Viability analysis performed on cortical neurons treated with 10 µM WZB117 or DMSO for 24 h. Statistical analysis ANOVA, followed by Tukey test. ** *p* < 0.01; *** *p* < 0.001. The data represent the means ± SEM of three experiments.

To confirm that WZB117 does not affect glial cell and GLUT3-positive neuron viability, we cultured U87 cells and primary astrocytes, which were treated with 5 and 10 μM WZB117 for 24 h. In U87 cells treated with 5 µM WZB117, no effect on viability was observed. In contrast, cells treated with 10 µM WZB117 had decreased viability by 28.57 ± 5.4% (Figure 6A). In the case of cortical astrocytes, a significant decrease in viability was observed from 10 µM (Figure 6B), which was close to 30%. DMSO did not alter cell viability (Figure 6B).

It has been proposed that WZB117 is a specific GLUT1 inhibitor, thus, when injected into the cerebral cortex, it should directly affect the entry of glucose and DHA into astrocytes. However, Ojelabi et al. [31] suggested that WZB117 could also bind GLUT3, a glucose transporter expressed at the neuronal level. Considering this information, we analyzed the effect of WZB117 treatment on neurons maintained in vitro under different conditions. In the first phase, cell viability was evaluated in the absence of glucose for 24 h, co-incubating with or without lactate, which can be used as an energy source in the absence of glucose (very abundant in the cerebral cortex at postnatal day 5). A 41.46% decrease was observed in cortical neurons in the absence of glucose, which was not recovered by lactate supplementation (Figure 6C). Subsequently, we evaluated neuronal viability in the presence of 10 µM WZB117, a concentration that was used in the in vivo injections, without observing a significant decrease in cell viability over 24 h (Figure 6D). Thus, neuronal viability in vitro is affected by glucose deprivation, a condition that is not generated by inhibiting GLUT1 with WZB117.

### 3.7. In Situ Inhibition of the DHA Recycling Transporter, GLUT1, Inhibited Neurite Formation in the Cerebral Cortex

**Figure 7:**
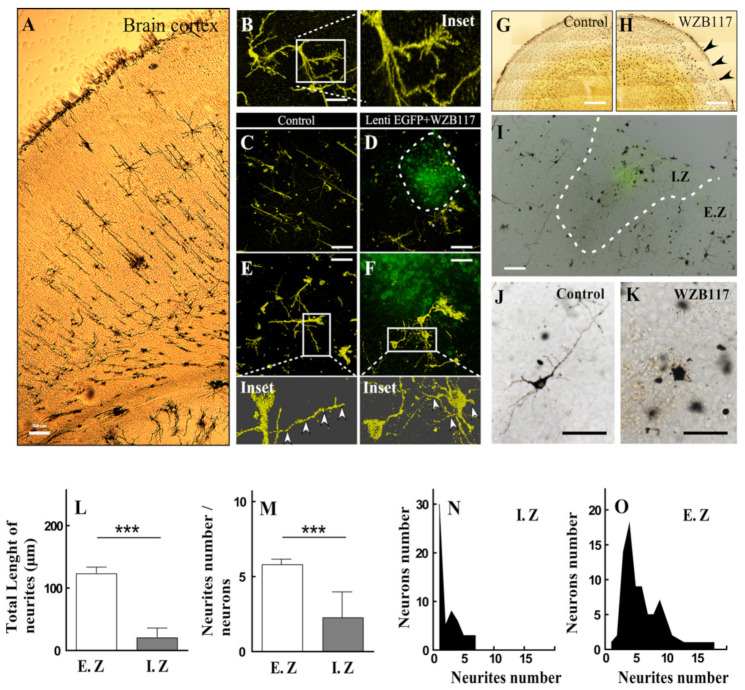
Golgi–Cox impregnation and confocal-reflection microscopy in postnatal rat cerebral cortex injected with lentivirus-EGFP and treated with WZB117. (**A**) Optic microscopy analysis of the outer cerebral cortex in 8 days of postnatal development rat brain after URG impregnation. (**B**) Confocal reflection microscopy (CRM) and 3D-Z-stack analysis of neuronal morphology of URG-impregnated neuronal cells. (**C**,**E**) Control cerebral cortex with URG-impregnation and CRM (**C**,**E** and inset). (**D**,**F**) Cerebral cortex treated with WZB117 and lentivirus-EGFP (depicted area in **D**) for 72 h and analyzed with URG-impregnation and CRM (**D**,**F** and inset). Neuronal neurites were analyzed with Z-stack-3D (Insets and arrows head). (**G**,**H**) Control rat brain (**G**) from 8 days of postnatal development or injected with 10 µM WZB117 for 72 h (**H**, arrows head) analyzed with impregnation with Golgi–Cox and Tile-scan reconstruction. (**I**) Brightfield microscopy analysis mixed with an epifluorescence image in the same area injected with lentivirus-EGFP and WZB117 (I. Z and depicted zone). The brain area most distant from the injection site was called E. Z. (**J**) Representative image of neurons present in the E. Z. (**K**) Representative image of neurons present in the I. Z. (**L**) Quantification of the total length of neurites quantified in neurons analyzed in the E. Z and I. Z. (**M**) Comparative analysis of the number of neurites quantified per neuron in the E. Z and I. Z. (**N**,**O**) Frequency graph of the number of neurites in the I. Z. and E. Z, respectively. Statistical analysis t-student (*** *p* < 0.001). Graph shows mean + SEM. In total, 30–40 neurons were quantified per area. Scale bars, in A, 500 µm; in (**B**), 15 µm; in (**C**,**D**), 250 µm; in (**E**,**F**), 150 µm; in (**G**,**H**), 1 mm; in I, 100 µm; in (**J**,**K**), 30 µm.

Rats at 8 days postnatal development were used to isolate the cerebral cortex and perform the URG silver impregnation technique (Figure 7A) in control animals or those 72 h-post-injected with EGFP lentivirus + WZB117 lentivirus (Figure 7C,D). Because the dark precipitate of this technique is composed of mercuric sulfide, the presence of this heavy metal allows its observation by confocal reflection microscopy, which additionally allows 3D reconstruction (Figure 7B and inset) for the analysis of primary and secondary neurites. Interestingly, despite the procedure performed during URG, the label for lentivirus-EGFP remained and was detected in the zone of co-administration with WZB117 (Figure 7D,F). 3D-reconstruction showed that the neurons of the control area present long processes (Figure 7E, inset and arrowhead), in contrast to neurons close to the injected area, where smaller extensions were detected (Figure 7F, inset and arrow head). Using confocal microscopy, analyses were performed using a bright field in order to quantify the morphological differences that could exist in the neuronal population (Figure 7G,H). Subsequently, epifluorescence microscopy was used to detect the EGFP-positive injected area (Figure 7I) and overlapped with the brightfield image of the section impregnated with URG (Figure 7I).

Using this superposition, the injected area (I.Z) and the area outside the injection (E.Z) were delimited (Figure 7I, depicted area). Neurons in the I.Z had a markedly lower presence of neuritic processes, compared to the neurons found in the E.Z (Figure 7J,K). Using the NeuroJ plugin (ImageJ software (W. Rasban, NIH), the presence and total length of neurites in the neurons present in the E.Z and I.Z were quantified. Thus, our analyses show that the neurons of the I.Z present a shorter length of neurites (20.14 ± 2.4 µm) compared to neurons of the E.Z (122.6 ± 9.7 µm) (Figure 7L). In addition, a smaller number of neurites was observed in the I.Z (Figure 7M). The above presented was observed in complementary graphs in N and O (Figure 7).

## 4. Discussion

In this work, we analyzed the formation of neurites in primary neurons that overexpress SVCT2 and in Neuro2a and HSVT-C3 neural cells. In addition, we used a 3D model of cell growth through the formation of neurospheres. Using SIM-SR microscopy analysis, we observed that SVCT2 is localized in the cell membrane in clusters. Furthermore, neuritic differentiation is finely regulated by the expression of SVCT2 and the intracellular concentration of vitamin C, which is maintained by a recycling mechanism that prevents DHA accumulation and generates an inverse effect in the differentiation process.

Although there are different studies that claim that vitamin C can stimulate neuronal differentiation of neurogenic precursors in brain development [7,32,33], we postulate that vitamin C is fundamentally related to the pluripotency properties of neural precursors because SVCT2 is preferentially expressed in radial glial cells and not in neuroblasts of the embryonic cortex [2]. In addition, differentiation of the cerebral cortex was not altered in SVCT2 knockout mice; however, postnatal functioning was affected [15]. Recently, different epigenetic functions have been described for vitamin C; it promotes the generation of mouse and human-induced pluripotent stem cells (iPSCs) [34,35] and cooperates with the histone demethylases, Jhdm1a/1b, to promote the reprogramming of somatic cells [36]. Vitamin C also enhances the expression of Nanog and inhibits retinoic acid-induced differentiation of embryonic stem cells [16,37]. Similarly, vitamin C treatment in neuronal cultures enhances the generation of midbrain-type dopamine neurons with improved survival and function of ventral midbrain-derived neural stem cells [6,38]. Although activation of the JAK/STAT/Nanog pathway by vitamin C and SVCT2 was initially associated with cellular pluripotency induction [16], a recent analysis in embryonal carcinoma F9 cells showed that SVCT2 functions as a receptor-like transporter of vitamin C, through the physical interaction between SVCT2 and JAK and the consequent activation of kinase in response to vitamin C [17].

Interestingly, activated JAK phosphorylates SVCT2, which, in turn, reinforces the activation of the JAK/STAT pathway promoting the following cellular responses: (i) inhibition of ROS generation; (ii) epigenetics regulation by promoting chromatin demethylation through the phosphorylation of demethylases and histone H3; (iii) transcription of pluripotency genes, directly by activating STAT and (iv) expression of neuronal differentiation genes by regulating some unknown effectors [17]. Additionally, entry of vitamin C into cells through the overexpression of SVCT1 does not activate the aforementioned pathway to stimulate differentiation [17]. A similar result was previously demonstrated in Neuro2a cells transduced with SVCT1-EYFP lentivirus, which increased vitamin C uptake but did not stimulate the formation of filopodia and processes [3]. In line with this notion, it would be expected that JAK/STAT signaling could be enhanced/facilitated with the oligomerization of SVCT2 transporters in the cell membrane. Analyses carried out in primary neuronal cells, Neuro2a and HSVT-C3 cells in this study illustrated SVCT2 clustering at the cell membrane, with an average size of approximately 130 nm. Considering the limitations of the analysis by SIM-super-resolution confocal microscopy, it would be expected that at least three transporters could be present in these clusters, probably making the transport of vitamin C and the activation of signaling pathways more efficient, such as JAK/STAT [17] and/or ERK1/2 [8].

We propose that the main function of vitamin C and SVCT2 is fundamentally to enhance neurite differentiation and inhibit ROS production in brain postnatal states [30,39]. In previous studies, we observed increased SVCT2 expression in pyramidal neurons of the cerebral and cerebellar cortex on postnatal days [1,8]. Using Neuro2a cells, we observed that the overexpression of SVCT2 generates an arborized phenotype with filopodia and neurites [3]. These results would represent postnatal stages of differentiation, likewise to the previously performed studies with in vitro neuronal differentiation [5,7,32].

By confocal analysis and SIM-SR microscopy, we observed that SVCT2 is mainly sorted with basolateral polarization, which is concentrated in vivo in dendritic-like projections but not in axonal-like MAP1B-positive structures. This confirms previous studies that identified a basolateral targeting sequence in the *N*-terminus of SVCT2, which is conserved among mammalian species [40]. Additionally, our data indicate that SVCT2 is localized in the cell membrane of dendritic-like projections that were PSD-95-positive, forming clusters. Even when large clusters of SVCT2 were detected in dendritic-like projections, co-localization with PSD95 was not observed. SVCT2 overexpression generates axonal-like MAP1B-positive structures, where pre-synaptic proteins, such as Piccolo, were observed in processes contacting “en passant” dendritic-like structures. Thus, SVCT2 overexpression increases the expression of proteins involved in neuronal differentiation and synapse formation, which are distributed in pre- and post-synaptic regions.

In vivo studies in postnatal guinea pigs subjected to a prolonged vitamin C diet deficiency, have impaired development of spatial memory [41], which involves synaptic plasticity events at the hippocampal level. All these antecedents suggest that AA, and its transporter, SVCT2, have an important role in the reorganization and appearance of new synapses in hippocampal neurons and, possibly in other regions of the CNS, such as the cerebral cortex. A recent study reported that vitamin C upregulated a series of mDA neuron-specific developmental and phenotype genes via suppression of DNA methylation and repressive histone code (H3K9m3, H3K27m3) at associated gene promoter regions [6].

Neurite formation in primary neurons was also replicated in Neuro2a and HSVT-C3 cells, which generated filopodia after overexpression of SVCT2. In these cells, overexpressed SVCT2 quickly reached the cell membrane due to the fact that a high colocalization was not observed with the RER. However, vesicular structures were observed in juxtapositions to the cell membrane that were positive for SVCT2-EYFP, or directly, inserted on the cell membrane. Furthermore, although SVCT2 can reach the mitochondria, colocalization between mitotracker and SVCT2-EYFP was not observed, which suggests that this functional property may be only associated with certain types of tumor cells [42]. Finally, it was evident that SVCT2 was observed in the different cells in cluster-like structures, which were detected in bleb- or ruffes-like membrane formations, or directly in filopodia.

When the nervous system uses vitamin C and differential expression of SVCT2 to stimulate neuritic growth, neurons increase their ability to concentrate AA, which rapidly oxidizes to DHA [20] and changes the metabolism of neural cells by inhibiting glycolysis, consuming glutathione and increasing the pentose pathway. DHA also increases lactate uptake by neural cells. Similar effects have recently been described in colon cancer cells treated with DHA or in cells of the immune system [28]. Under oxidant generation conditions, DHA accumulation induces cell death, which can be avoided for DHA recycling by astrocytes that express GLUT1, and actively capture this molecule [19]. Intracellularly, DHA would be partially reduced within the neuron in the cytosol, by means of GSH [20]. DHA could also be compartmentalized in the endoplasmic reticulum by means of GLUT10 [22]; however, this pathway is unlikely in neurons, since DHA may induce ER stress [43] and neurons do not express GLUT10. Reduced vitamin C (ascorbic acid) is more likely to be transported within the ER [44], since AA is a co-factor of enzymes such as iron (Fe^2+^) and α-KG -dependent dioxygenases (Fe^2+^/α-KGDD). Inside ER, the DHA generated for the aforementioned enzymatic reactions would be reduced by PDI; however, this reaction is too slow to be the major route for a reduction in DHA in the ER [45].

If the recycling systems are bypassed and DHA accumulates in neurons, a non-apoptotic type of death is induced, called necroptosis [21]. In line with this notion, vitamin C also regulates the expression of RIPK1/MLKL, whereas the oxidation of AA in neurons induces morphological alterations consistent with necroptosis and MLKL activation. Activation of necroptosis by AA oxidation in neurons results in bubble formation, loss of membrane integrity, and ultimately, cellular explosion. Finally, generation of MLKL^−/−^ and SVCT2^−/−^ neuronal cells by CRISPR/Cas9 delayed cell death. Furthermore, inhibition of AA uptake by the deletion of SVCT2 completely prevented neuronal death, suggesting that oxidation of vitamin C and generation of DHA could regulate neuronal necroptosis under conditions of oxidative stress [21].

During cerebral cortex maturation, the “bystander effect” [46] may be an efficient mechanism to inhibit metabolic changes in neurons, which may affect their differentiation and finally induce cell death [19,21]. Thus, it is essential that DHA be recycled between neurons and astrocytes. Interestingly, only neurons increase SVCT2 expression at the postnatal stage; therefore, astrocytes do not uptake AA. Neuritic arborization begins in postnatal developmental stages and in the brain regions that have generated functional astrocytes [47]. Of note, TH-positive dopaminergic cells grafted inside the brain formed by vitamin C-treated neural stem cells were abundantly surrounded by GFAP-positive astrocytes [6]. In this sense, we cannot ignore that transplantation of astrocytes alone or together with neurogenic donor cells has become a therapeutic possibility for treating brain disorders [6,48,49,50].

In this work, we inhibited GLUT1 in vivo in the cerebral cortex. We assume that the effect was fundamentally generated in astrocytes, inducing locally and for a certain time, DHA-recycling inhibition between neurons and astrocytes. We postulate that this is possible by the following reasoning. If the neurons and astrocytes of the cerebral cortex were totally dependent on glucose uptake, as the single source of energy, the presence of WZB117 inside the cortex, would affect their viability through deprivation of glucose uptake. However, it has been proposed that during the first postnatal weeks the brain is nourished mainly by ketone bodies, obtained from breast milk [51]; therefore, both astrocytes and neurons would not depend on glucose uptake for maintenance of metabolism. Thus, WZB117 should fundamentally affect DHA uptake to astrocytes, and not to neurons, which should also express GLUT3. Finally, we are aware that testing vitamin C recycling in vivo is a complex challenge; however, this study may represent a valid approach to begin to validate this recycling mechanism inside the brain.

## 5. Conclusions

We observed that neurons overexpressing SVCT2 have increased neuritic arborization; however, higher doses of reduced vitamin C significantly reduced the formation of neurites when the neurons were not incubated with a bystander DHA recycling cell, such as U87 cells. Additionally, the results strongly suggested that the presence of a cell that efficiently recycles DHA, from the neurospheres culture medium, was necessary for the maintenance of neuritic growth. Furthermore, inhibition of GLUT1 in an astrocyte-like cell, preventing the recycling of DHA, in turn, inhibited the growth of neurites. Finally, the use of URG impregnation demonstrated that the presence of an inhibitor of DHA uptake (through GLUT1) in astrocytes decreased neurite length and arborization.

## Data Availability

The data presented in this study are available in this manuscript.
